# Dissemination and evaluation of the ASAS/EULAR recommendations for the management of ankylosing spondylitis: results of a study among 1507 rheumatologists

**DOI:** 10.1136/ard.2007.080077

**Published:** 2008-11-29

**Authors:** L Gossec, M Dougados, C Phillips, M Hammoudeh, K de Vlam, K Pavelka, T Pham, J Braun, J Sieper, I Olivieri, D van der Heijde, E Collantes, M Stone, T K Kvien, on behalf of ASAS (ASsessment in AS international working group)

**Affiliations:** 1 Paris Descartes University, Medicine Faculty; UPRES-EA 4058; APHP, Rheumatology B Department, Cochin Hospital, Paris, France; 2 Wyeth Europa, UK; 3 Hamad Medical Corporation, Doha, Qatar; 4 Rheumatology, University Hospital Leuven, Leuven, Belgium; 5 Institute of Rheumatology and Clinic of Rheumatology Charles University, Prague, Czech Republic; 6 Rheumatology Department, Conception Hospital, AP-HM, Marseille, France; 7 Rheumazentrum Ruhrgebiet Herne and Ruhr University, Bochum, Germany ^8^Rheumatology, Medical Department I, Charité, Campus Benjamin Franklin, Berlin, Germany; 8 Rheumatology Department of Lucania, San Carlo Hospital of Potenza and Madonna delle Grazie Hospital of Matera, Matera, Italy; 9 Leiden University Medical Centre, Leiden, The Netherlands; 10 University Hospital Reina Sofía, Cordoba, Spain; 11 University of Bath, UK and University of Toronto, Canada; 12 Department of Rheumatology, Diakonhjemmet Hospital, Oslo, Norway

## Abstract

**Background:**

Ten ASAS/EULAR recommendations for the management of ankylosing spondylitis (AS) were published in 2006.

**Objectives:**

(*a*) To disseminate and (*b*) to evaluate conceptual agreement with, and (*c*) application of, these recommendations as well as (*d*) potential barriers to the application.

**Methods:**

A questionnaire was sent to rheumatologists in 10 countries. It included (*a*) the text of the recommendations; (*b*) rheumatologists’ demographic variables; (*c*) two numerical rating scales from 1 to 10 for each recommendation: conceptual agreement with, and application of, the recommendation (10 indicates maximal agreement and maximal application); and (*d*) a list of potential barriers to the application of the recommendation. Statistical analysis included descriptive and multivariate analyses.

**Results:**

7206 questionnaires were sent out; 1507 (21%) were returned. Of the 1507 answering rheumatologists, 62% were men, mean (SD) age 49 (9) years, and 34% had an academic position. Conceptual agreement with the recommendations was high (mean (SD) for all recommendations 8.9 (0.9)). Self-reported application was also high (8.2 (1.0)). The difference between agreement and application varied across recommendations and countries. The most pronounced discrepancies were reported for use of anti-tumour necrosis factor drugs in a few countries, with funding as the most commonly reported barrier for application of this recommendation.

**Conclusion:**

This large project has helped the dissemination of the ASAS/EULAR recommendations for the management of AS and shows that conceptual agreement with the recommendations is very high. The project also highlights inequalities in access to healthcare for European citizens with AS.

In 2006, recommendations for the management of ankylosing spondylitis (AS) were published under the umbrella of the ASsessment in AS international working group (ASAS), and the European League Against Rheumatism (EULAR).^1^ These recommendations concern all aspects of management of AS (box 1). The Institutes of Medicine define clinical practice guidelines or recommendations as “systematically developed statements to assist practitioner and patient decision about appropriate health care for specific clinical circumstances”. Clinical guidelines may induce small improvements, both in processes and in the outcomes of care.^2^ However, if recommendations are to have effect, it is necessary, after having published them, to facilitate their dissemination.^3^ Simple top-down dissemination of monodisciplinary guidelines alone is not effective.^4^^–^8 A more powerful strategy to change behaviour is to involve doctors directly.^9^ Implementation experts indicate that multistage involvement in the development of a guideline can be a positive contributor to effective implementation of guidelines.^10^ ^11^ Therefore, an ASAS-initiated project was performed in 2006, involving practising rheumatologists in 10 countries.

The objectives of this study were (*a*) to disseminate and (*b*) to evaluate conceptual agreement with, and (*c*) self-reported application as well as (*d*) potential barriers to the application of, the ASAS/EULAR recommendations among rheumatologists from 10 different countries.

**Box 1: ASAS/EULAR recommendations for the management of ankylosing spondylitis (AS)^1^**

1. Treatment of AS should be tailored according to current manifestations of the disease (axial, peripheral, entheseal, extra-articular symptoms and signs), level of current symptoms, clinical findings and prognostic indicators: disease activity/inflammation; pain; function, disability, handicap; structural damage, hip involvement, spinal deformities; general clinical status (age, sex, comorbidity, concomitant drugs); wishes and expectations of the patient.
2. Disease monitoring of patients with AS should include a patient history (eg, questionnaires), clinical measures, laboratory tests and imaging, all according to the clinical presentation, as well as the ASAS core set*. The frequency of monitoring should be decided on an individual basis depending on symptoms, severity and drug treatment.
3. Optimal management of AS requires a combination of non-pharmacological and pharmacological treatments.
4. Non-pharmacological treatment of AS should include patient education and regular exercise. Individual and group physical therapy should be considered and patient associations and self-help groups may be useful.
5. Non-steroidal anti-inflammatory drugs (NSAIDs) are recommended as first-line drug treatment for patients with AS who have pain and stiffness. In those with increased gastrointestinal risk, non-selective NSAIDs plus a gastroprotective agent, or a selective COX-2 inhibitor could be used.
6. Analgesics, such as paracetamol and opioids, might be considered for pain control in patients in whom NSAIDs are insufficient, contraindicated and/or poorly tolerated.
7. Corticosteroid injections directed to the local site of musculoskeletal inflammation may be considered. The use of systemic corticosteroids for axial disease is not supported by evidence.
8. There is no evidence for the efficacy of disease-modifying antirheumatic drugs (DMARDs), including sulfasalazine and methotrexate, for the treatment of axial disease. Sulfasalazine may be considered in patients with peripheral arthritis.
9. Anti-tumour necrosis factor (TNF) treatment should be given to patients with persistently high disease activity despite conventional treatments according to the ASAS recommendations. There is no evidence to support the obligatory use of DMARDs before, or concomitant with, anti-TNF treatment in patients with axial disease.
10. Total hip arthroplasty should be considered in patients with refractory pain or disability and radiographic evidence of structural damage, independent of age. Spinal surgery—for example, corrective osteotomy and stabilisation procedures— may be of value in selected patients.

*The ASAS core set includes domains on axial, peripheral and enthesopathological manifestations. One or more specific instruments are recommended for each domain.

## METHODS

This project was initiated by ASAS, supervised by Maxime Dougados (France) and Tore K Kvien (Norway) and financially supported by Wyeth Europa pharmaceutical company.

A questionnaire was prepared which included the text of the recommendations, demographic variables (age, sex, academic position or not, number of years of practice and mean number of rheumatic patients and patients with AS seen a month) and a numerical rating scale (NRS) from 1 to 10 for conceptual agreement and application of each recommendation (10 indicated maximal agreement and maximal application). The text was: “Do you conceptually agree with this recommendation?” and “Are you applying this recommendation in your daily practice?”. The questionnaire also included a list of potential barriers to the application of the recommendation (the rheumatologist could tick as many barriers as applicable). The barriers were different for each recommendation and were selected by the authors on the basis of clinical experience. Respondents could also volunteer additional barriers.

Ten countries participated: Arabian Gulf, Belgium, Czech Republic, France, Germany, Italy, the Netherlands, Norway, Spain and the United Kingdom (UK). For each country, a national investigator (member of ASAS) translated the questionnaire and sent it out to rheumatologists between March and September 2006. The list of rheumatologists who would receive the questionnaire was freely chosen by the national investigator. The rheumatologists were asked to complete and return the questionnaire to the national investigator.

Statistical analysis was performed by LG on anonymous data with knowledge of country; analysis was descriptive for conceptual agreement and self-reported application. Multivariate logistic regression analyses were also performed. In the first, the dependent variable was conceptual agreement per doctor, binarised at the mean conceptual agreement for that recommendation. In the second analysis the dependent variable was the individual difference between agreement and application (ie, agreement minus application for a given doctor), also binarised at the mean. The independent variables entered in both analyses were country and the characteristics of the rheumatologist. Statistical analyses were performed with SAS version 9.0.

## RESULTS

### Response rate and participants

A total of 7206 questionnaires were sent out. The response rate varied across countries from 49% (Italy) to 11% (Spain), but the number of mailed questionnaires also varied widely between countries (table 1). Thus, the total number of questionnaires returned and analysed was 1507 (21% of all questionnaires), of which 413 (27%) of all analysed questionnaires were from France and 301 (20%) were from Germany (table 1).

**Table 1:** Response rate and characteristics of the 1507 European rheumatologists who were respondents to the questionnaire evaluating the ASAS/EULAR recommendations for the management of ankylosing spondylitis (AS)

Characteristics		Arabian Gulf (n = 89)	Belgium (n = 84)	Czech Republic (n = 73)	France (n = 413)	Germany (n = 301)	Italy (n = 172)	Netherlands (n = 73)	Norway (n = 72)	Spain (n = 119)	UK (n = 111)	All countries (n = 1507)
Respondents (%)		28.5	28.8	20.9	16.0	27.0	49.1	30.2	36.0	10.9	17.3	20.9
Distribution across countries (% of all respondents)		5.9	5.6	4.8	27.4	20.0	11.4	4.8	4.8	7.9	7.4	100
Men (%)		79.8	62.2	27.4	60.1	63.1	68.6	65.8	47.2	60.5	75.7	62.1
Age (years), mean (SD)		46.4 (6.5)	49.1 (10.8)	47.8 (8.8)	48.3 (8.9)	50.9 (8.5)	48.7 (10.2)	51.1 (7.7)	50.0 (9.5)	46.8 (9.3)	45.8 (8.4)	48.6 (9.1)
Number of years of practice, mean (SD)		14.1 (7.6)	18.3 (10.7)	15.8 (7.9)	18.5 (8.7)	15.6 (9.6)	17.6 (9.9)	16.0 (8.6)	14.1 (9.2)	18.0 (9.9)	14.7 (8.3)	16.8 (9.3)
Academic position (%)		65.2	27.4	19.2	13.1	77.7	22.1	19.2	25.0	28.6	19.8	33.8
Number of rheumatic patients seen a month, mean (SD)		188.9 (133.1)	220.3 (126.4)	268.6 (192.9)	275.1 (159.9)	224.7 (202.7)	160.8 (120.5)	277.7 (162.7)	123.7 (222.2)	302.1 (143.7)	217.6 (153.7)	234.4 (171.7)

Range		8–800	3–500	20–750	20–1200	5–1000	5–800	40–850	0–1800	40–850	10–1250	0–1800
Number of patients with AS seen per month, mean (SD)		10.1 (32.7)	15.4 (13.5)	16.8 (16.7)	12.0 (25.9)	25.0 (43.3)	9.9 (12.5)	23.9 (61.1)	12.7 (10.0)	18.4 (16.6)	12.9 (9.8)	15.6 (29.8)

Range		1–300	1–50	2–70	1–415	1–600	1–100	1–510	0–40	1–100	2–60	0–600

Table 1 shows the characteristics of the rheumatologists. Of respondents, 62% were men, mean (SD) age 49 (9) years, mean (SD) years of practice 17 (9), and 34% reported having an academic position. The mean (SD) number of patients seen a month was 234 (172) and the mean (SD) number of patients with AS seen a month was 15.6 (29.8).

### Conceptual agreement

Conceptual agreement with the recommendations was evaluated separately for each recommendation, and was generally high. Mean (SD) for all recommendations and all countries was 8.9 (0.9) (table 2), and more than 80% of all rheumatologists had an agreement ⩾7 for all recommendations combined (table 2). Agreement was highest for recommendation 3 (“optimal management of AS requires a combination of non-pharmacological and pharmacological treatments”) and recommendation 1 (“tailoring of treatment”) (mean (SD) 9.5 (1.1) and 9.3 (1.2), respectively). Agreement was lowest for recommendation 6 (“analgesics, such as paracetamol and opioids, might be considered”) with a mean (SD) value of 8.3 (2.0).

**Table 2:** Scores for self-reported conceptual agreement with each ASAS/EULAR recommendation, scores for self-reported application of each recommendation and difference between conceptual agreement and self-reported application (agreement – application) of each recommendation across countries

Recommendation	Agreement All countries	Application All countries	Difference between agreement and application										
			Arabian Gulf	Belgium	Czech R.	France	Germany	Italy	Netherlands	Norway	Spain	UK	All countries
1	9.3 (1.2)	8.7 (1.4)	0.9 (1.2)	0.3 (1.6)	1.1 (1.4)	0.3 (1.2)	0.5 (1.0)	0.6 (1.4)	0.5 (1.6)	0.8 (1.5)	0.7 (1.1)	1.0 (2.0)	0.6 (1.4)
	[97.2]	[93.4]											[36.2]
	10 (1–10)	9 (1–10)											0 (–9 to 9)
2	8.9 (1.5)	7.7 (2.0)	1.2 (1.5)	1.3 (2.4)	1.2 (1.4)	0.8 (1.4)	0.6 (1.2)	1.4 (1.6)	1.6 (1.9)	1.2 (1.9)	1. (1.6)	2.0 (1.9)	1.1 (1.6)
	[92.5]	[75.6]											[51.1]
	9 (1–10)	8 (1–10)											1 (−8 to 9)
3	9.5 (1.1)	8.8 (1.5)	1.3 (1.7)	0.3 (1.7)	0.3 (0.9)	0.5 (1.3)	0.6 (1.2)	1.1 (1.4)	0.2 (0.5)	0.7 (1.2)	1.1 (1.6)	0.9 (1.6)	0.7 (1.4)
	[97.7]	[91.1]											[35.0]
	10 (1–10)	9 (2–10)											0 (–9 to 8)
4	8.9 (1.6)	7.5 (2.1)	1.9 (2.1)	1.0 (1.6)	1.0 (1.4)	1.4 (1.7)	1.1 (1.7)	2.2 (2.1)	0.5 (0.9)	0.9 (1.0)	1.9 (2.0)	1.5 (1.8)	1.4 (1.8)
	[90.8]	[69.7]											[56.5]
	10 (1–10)	8 (1–10)											0 (−9 to 9)
5	9.2 (1.4)	8.9 (1.5)	0.3 (0.8)	0.7 (2.0)	0.4 (1.0)	0.1 (0.7)	0.4 (0.9)	0.2 (0.8)	0.1 (0.6)	0.4 (0.8)	0.2 (0.7)	0.2 (1.1)	0.3 (0.9)
	[94.5]	[92.4]											[19.7]
	10 (1–10)	9 (1–10)											0 (−9 to 7)
6	8.3 (2.0)	7.8 (2.3)	0.7 (1.4)	0.5 (1.6)	1.0 (1.5)	0.3 (1.1)	0.6 (1.2)	0.7 (1.1)	0.6 (1.1)	0.9 (1.2)	0.6 (1.1)	0.4 (0.9)	0.5 (1.2)
	[81.8]	[73.9]											[32.5]
	9 (1–10)	8 (1–10)											0 (−9 to 7)
7	8.6 (1.8)	8.1 (2.1)	0.6 (1.1)	0.4 (1.7)	0.5 (1.3)	0.4 (1.2)	0.8 (1.5)	0.9 (1.6)	0.5 (1.2)	0.7 (1.1)	0.7 (1.4)	0.6 (1.1)	0.6 (1.4)
	[87.5]	[79.6]											[32.1]
	9 (1–10)	9 (1–10)											0 (−8 to 9)
8	8.6 (1.9)	8.3 (2.1)	0.3 (1.2)	0.2 (2.1)	0.3 (0.7)	0.3 (1.1)	0.5 (1.1)	0.2 (1.1)	0.0 (1.0)	0.5 (0.9)	0.4 (1.3)	0.2 (1.0)	0.3 (1.2)
	[79.7]	[82.7]											[22.8]
	9 (1–10)	9 (1–10)											0 (−9 to 9)
9	9.0 (1.6)	8.0 (2.3)	1.6 (2.3)	0.4 (1.8)	2.7 (2.9)	0.6 (1.3)	0.9 (1.8)	0.9 (1.9)	0.4 (0.9)	0.8 (1.2)	0.8 (1.5)	2.8 (3.0)	1.0 (2.0)
	[96.6]	[79.1]											[37.8]
	10 (1–10)	9 (1–10)											0 (−9 to 9)
10	8.6 (1.7)	7.8 (2.2)	1.2 (1.6)	0.7 (1.5)	1.1 (1.7)	0.6 (1.4)	0.7 (1.4)	1.4 (1.9)	0.6 (1.0)	0.6 (1.2)	1.0 (1.5)	0.5 (1.0)	0.8 (1.5)
	[87.1]	[74.5]											[36.7]
	9 (1–10)	8 (1–10)											0 (−9 to 9)
Mean	8.9 (0.9)	8.2 (1.0)	0.9 (0.8)	0.5 (1.3)	0.9 (0.8)	0.5 (0.7)	0.7 (0.7)	1.0 (0.8)	0.5 (0.5)	0.8 (0.7)	0.9 (0.7)	1.0 (0.9)	0.7 (0.8)
	[90.8]	[81.7]											[30.2]
	9 (1–10)	8 (1–10)											0.6 (−6.6 to 4.4)

See box 1 for the text of the recommendations.

Agreement (first column): the question was: “Do you conceptually agree with this recommendation?” (1–10 scale where 10 indicates high agreement). Results are presented for all countries (pooled results) as mean (SD) agreement with recommendation, [% rheumatologists with an agreement ⩾7], median (range).

Application (second column): The question was: “Are you applying this recommendation in your daily practice?” (1–10 scale where 10 indicates high application). Results are presented for all countries (pooled results) as mean (SD) self-declared application of recommendation, [% rheumatologists with an application ⩾7], median (range).

Individual difference (agreement-application): results are presented separately for each country and (last column) as pooled results, as mean (SD). Last column also shows [% rheumatologists with a difference ⩾1] and median (range).

Agreement varied across countries; mean agreement with all recommendations was highest in the Czech Republic (mean (SD) 9.4 (0.5)) and in Norway (mean (SD) 9.2 (0.6)) and was lowest in Belgium (mean (SD) 8.5 (1.4)).

Multivariate analyses were performed to explain agreement with the recommendations. For all recommendations pooled, the country of the investigator was not statistically associated with agreement. Only female gender (odds ratio (OR) = 1.33, 95% confidence interval (CI) 1.03 to 1.72, p = 0.028) and academic position (OR = 1.31, 95% CI 1.02 to 1.70, p = 0.032) were predictive of higher agreement. However, this statistical significance did not reflect clinical relevance. Mean (SD) agreement scores were 8.93 (0.930) versus 8.84 (0.90) for female versus male rheumatologists, and 8.95 (0.81) versus 8.80 (0.96) for academics versus non-academics, thus high for all doctors.

### Application of recommendations

Self-declared application of recommendations was also high, but lower than conceptual agreement (mean (SD) 8.2 (1.0)) (table 2). Application scores higher than 7 were reported by 81.7% of the doctors. Self-reported application was highest for recommendation 5 (“non-steroidal anti-inflammatory drugs (NSAIDs) are recommended as first-line drug treatment”) with mean (SD) score 8.9 (1.5). Self-reported application was lowest for recommendation 4 (“non-pharmacological treatment of AS should include patient education and regular exercise. Individual and group physical therapy should be considered and patient associations and self-help groups may be useful”) and recommendation 2 (“disease monitoring of patients with AS”) with mean (SD) scores of 7.5 (2.1) and 7.7 (2.0), respectively.

Application varied across countries; mean (SD) self-reported application of all recommendations was highest in the Czech Republic (8.5 (0.9)) and was lowest in the UK (7.8 (0.9)).

### Difference between agreement and self-reported application

We calculated, for each recommendation, the difference for each doctor between self-declared agreement and application—that is, agreement minus application. This score could range from 0 (no difference between agreement and application) to 10 (total agreement but no application). Theoretically, negative scores could also be obtained (if application was higher than agreement).

The differences between agreement and application varied across recommendations and across countries (table 2). The difference between agreement and application had mean values above 1.0 for three recommendations. The highest values were 1.4 (1.8), for recommendation 4 (“non-pharmacological treatment of AS”), 1.1 (1.6), for recommendation 2 (“disease monitoring of patients with AS”) and 1.0 (2.0), for recommendation 9 (“anti-tumour necrosis factor (TNF)”). The lowest values were observed for recommendations 5 and 8 (mean 0.3 (0.9) and 0.3 (1.2), respectively). There were no negative scores.

The largest overall country differences between agreement and application were recorded in Italy (1.0 (0.8)) and the UK (1.0 (0.9)). For recommendation 4, mean difference scores were >1 for all countries except the Netherlands and Norway, but were >2 only for Italy. For recommendation 2, mean difference scores were >1 for all countries except France and Germany, but were >2 only for the UK. For recommendation 9 (“anti-TNF”), mean difference scores were <1 for all countries except UK (2.8 (3.0)), Czech Republic (2.7 (2.9)) and the Arabian Gulf (1.6 (2.3)).

Multivariate analyses were performed to explain this difference between agreement and application. Country was the only independent variable which was significantly associated with the difference between agreement and application (p<0.001) (data not shown).

### Barriers to application of recommendations

Table 3 shows items presented as potential barriers and ticked by more than 25% of rheumatologists. A high proportion of doctors felt there was no specific barrier to the application of recommendations 1, 5, 6, 7, 8 and 10 (more than 40% ticked the item “there is no specific barrier”). The potential presence of barriers for application was most frequently reported for recommendation 4 (no barrier, 19.7%), 2, 3 and 9 (no barrier, 30.3%, 32.0% and 34.5%, respectively).

**Table 3:** Barriers to application of recommendations

Recomm.	Item		Arabian Gulf	Belgium	Czech R.	France	Germany	Italy	Netherlands	Norway	Spain	UK	All countries
1	There is no barrier		29 (32.6)	37 (44.1)	23 (31.5)	234 (56.7)	151 (50.2)	66 (38.4)	31 (42.5)	31 (43.1)	53 (44.5)	22 (19.8)	677 (44.9)
	Consultation time to conduct assessment fully		21 (23.6)	22 (26.2)	19 (26.0)	55 (13.3)	72 (23.9)	47 (27.3)	33 (45.2)	20 (27.8)	59 (49.6)	45 (40.5)	393 (26.1)
													
2	There is no barrier		16 (18.0)	20 (23.8)	17 (23.3)	145 (35.1)	140 (46.5)	46 (26.7)	16 (21.9)	25 (34.7)	18 (15.1)	13 (11.7)	456 (30.3)
	Lack of time to conduct frequent monitoring		33 (37.1)	42 (50.0)	33 (45.2)	NA	58 (19.3)	68 (39.5)	38 (52.1)	29 (40.3)	84 (70.6)	79 (71.2)	464 (42.4)*
													
3	There is no barrier		28 (31.5)	33 (39.3)	13 (17.8)	138 (33.4)	107 (35.6)	32 (18.6)	50 (68.5)	30 (41.7)	26 (21.9)	25 (22.5)	482 (32.0)
	Insufficient number of qualified health professionals—eg, physiotherapists		23 (25.8)	11 (13.1)	18 (24.7)	70 (16.9)	44 (14.6)	97 (56.4)	4 (5.5)	12 (16.7)	67 (56.3)	45 (40.5)	391 (25.9)
	Lack of facilities for education		19 (21.4)	10 (11.9)	19 (26.0)	122 (29.5)	59 (19.6)	62 (36.1)	2 (2.7)	11 (15.3)	60 (50.4)	14 (12.6)	378 (25.1)
													
4	There is no barrier		10 (11.2)	16 (19.1)	16 (21.9)	72 (17.4)	79 (26.25)	14 (8.14)	34 (46.6)	23 (31.9)	9 (7.6)	24 (21.6)	297 (19.7)
	Lack of patient compliance with recommendations		49 (55.1)	51 (60.7)	47 (64.4)	214 (51.8)	121 (40.2)	74 (43.0)	24 (32.9)	28 (38.9)	63 (52.9)	39 (35.1)	710 (47.1)
	Lack of facilities for education		36 (40.5)	18 (21.4)	21 (28.8)	158 (38.3)	99 (32.9)	59 (34.3)	4 (5.5)	15 (20.8)	60 (50.4)	26 (23.4)	496 (32.9)
	Insufficient number of qualified health professionals—eg, physiotherapists		35 (39.3)	16 (19.1)	28 (38.4)	73 (17.7)	55 (18.3)	104 (60.5)	4 (5.5)	17 (23.6)	67 (56.3)	56 (50.5)	455 (30.2)
													
5	There is no barrier		42 (47.2)	10 (11.9)	30 (41.1)	223 (54.0)	141 (46.8)	44 (25.6)	42 (57.5)	12 (16.7)	46 (38.7)	38 (34.2)	628 (41.7)
	Concerns about the safety of long-term use of NSAIDs/COX-2 inhibitor		34 (38.2)	50 (59.5)	37 (50.7)	117 (28.3)	95 (31.6)	73 (42.4)	17 (23.3)	42 (58.3)	48 (40.3)	57 (51.4)	570 (37.8)
													
6	There is no barrier		29 (32.6)	17 (20.2)	25 (34.3)	223 (54.0)	160 (53.2)	79 (45.9)	41 (56.2)	11 (15.3)	64 (53.8)	65 (58.6)	714 (47.4)
	Fear of addiction/tolerance to opioids with long-term use		38 (42.7)	50 (59.5)	35 (47.9)	99 (24.0)	61 (20.3)	46 (26.7)	14 (19.2)	51 (70.8)	25 (21.0)	31 (27.9)	450 (29.9)
													
7	There is no barrier		37 (41.6)	37 (44.1)	40 (54.8)	182 (44.1)	149 (49.5)	64 (37.2)	45 (61.6)	29 (40.3)	59 (49.6)	66 (59.5)	708 (47.0)
	Patient concerns about use of corticosteroid injections		43 (48.3)	31 (36.9)	27 (37.0)	157 (38.0)	78 (25.9)	55 (32.0)	11 (15.1)	25 (34.7)	36 (30.3)	21 (18.9)	484 (32.1)
													
8	There is no barrier		46 (51.7)	42 (50.0)	47 (64.4)	255 (61.7)	175 (58.1)	97 (56.4)	54 (74.0)	35 (48.6)	70 (58.8)	66 (59.5)	887 (58.9)
													
9	There is no barrier		21 (23.6)	24 (28.6)	5 (6.9)	175 (42.4)	119 (39.5)	55 (32.0)	43 (58.9)	22 (30.6)	49 (41.2)	13 (11.7)	526 (34.9)
	Insufficient funding		41 (46.1)	6 (7.1)	57 (78.1)	32 (7.8)	102 (33.9)	38 (22.1)	3 (4.1)	19 (26.4)	20 (16.8)	73 (65.8)	391 (25.9)
	Administrative burden associated with anti-TNF treatment		26 (29.2)	24 (28.6)	24 (32.9)	101 (24.5)	48 (16.0)	74 (43.0)	13 (17.8)	9 (12.5)	39 (32.8)	28 (25.2)	386 (25.6)
													
10	There is no barrier		27 (30.3)	26 (30.9)	23 (31.5)	193 (46.7)	184 (61.1)	61 (35.5)	48 (65.8)	34 (47.2)	44 (36.9)	68 (61.3)	708 (47.0)

Items ticked by more than 25% of rheumatologists are presented.

For each item: number (percentage) where the item was ticked.

*Percentage of available data.

NA, not available, NSAIDs, non-steroidal anti-inflammatory drugs; Recomm., recommendation; TNF, tumour necrosis factor.

The potential barrier “I am not familiar with this recommendation” was included in the questionnaire for all recommendations but was infrequently ticked (0.2% to 6% for all recommendations).

For recommendations 1 and 2, the most frequent barrier (26.1% and 42.4% of doctors, respectively) was “lack of time”. More than 70% of doctors in Spain and the UK reported this barrier for recommendation 2. For recommendation 4, the most frequent barrier (47.1% of doctors) was “lack of patient compliance with recommendations”. The second most frequent barrier (32.9%) was “lack of facilities for education”, particularly in Spain (50.4%). The most frequent barrier (37.8%) for recommendation 5 was “concerns about the safety of long-term use of NSAIDs/COX-2 inhibitors”, but 41.7% reported no specific barrier. For recommendation 6, 47.4% of doctors reported no specific barrier; but 29.9% reported that “fear of addiction/tolerance to opioids with long-term use” was a barrier. The most frequent barrier (32.1%) for recommendation 7 was “patient concerns about use of corticosteroid injections” but 47.0% of doctors reported no specific barrier for the application of this recommendation.

For recommendation 9 regarding use of anti-TNF drugs in patients with AS, the most frequent barriers were “insufficient funding” (25.9%), and “administrative burden associated with anti-TNF treatment” (25.6%). Major differences across countries were observed for this item. Barriers related to funding were frequently reported in the Czech Republic (78.1%) and the UK (65.8%), whereas Italian doctors frequently reported barriers related to administrative burden (43.0%).

## DISCUSSION

This large project has helped the dissemination of the ASAS/EULAR recommendations for the management of AS: 7206 rheumatologists received the questionnaire and thus also the text of the recommendations, and 1507 answered and are assumed to have at least read the recommendations. The results from the survey showed that conceptual agreement with the recommendations was very high (mean 8.9), and self-declared application was also high, though somewhat lower (mean 8.2).

Raising implementation of recommendations is not an easy task. Implementation experts indicate that the effect of issued recommendations on clinical practice is improved if practising clinicians actively contribute to the development of the recommendations.^12^ If this involvement is not feasible, creation of a sense of “ownership” through a process of dissemination is essential for successful implementation of recommendations in clinical practice.^10^ ^12^ We have paid specific attention to these aspects, first, by involving worldwide representation of rheumatologists in the elaboration 1, and second, by focusing specifically on the doctors’ acceptance of the recommendations by including them in the validation procedure, and giving them the opportunity to express their agreement and practical applicability of each recommendation.

Agreements with the 10 recommendations were generally high. The ASAS group is possibly considered as an opinion leader to such extent that the rheumatologists did not feel justified, or diplomatic, to disagree with recommendations that were prepared by experts they trusted. Another factor that might have had an impact on the validity of the results is the method of data collection. Some authors have recommended feedback meetings rather than questionnaires as an effective method to create involvement among doctors during a validation procedure.^9^ However, meetings are time consuming and costly and their impact may be only moderate.^9^ Furthermore, some doctors might have had comments that were not expressed since they considered that their input would not have any impact in the final recommendations. Another potential explanation is related to selection of respondents by their agreement. This is a possible bias which needs to be discussed because the response rate (21%) is very low, and the percentage of academic doctors is high (34%). However, it should be noted that these response rates in the context of an implementation project were reasonable. Response rates varied across countries, and were higher in countries where fewer rheumatologists were solicited, presumably because in these countries a selection was operated before sending the questionnaires—that is, these doctors were all treating patients with AS and wanted to answer. The reason for this low response rate, in particular, in certain countries is probably a suboptimal selection of the rheumatologists receiving the questionnaires. The list of recipients was freely determined by the principal investigator for each country, and in some countries a national society of rheumatology members’ list was used. This selection method may be insufficiently directed towards interested rheumatologists, involved with patients with AS, which may explain the low response rate. It also may explain why some of the responding rheumatologists reported having a small number of patients with AS.

The application of recommendations for management is generally important for the quality of patient care. Self-reported application of these recommendations was generally high across Europe. The implementation of guidelines and recommendations remains a challenge world wide, as barriers exist at several levels. These barriers may be generic, such as national limitations to prescriptions of expensive drugs or a general lack of adequate resources and/or poor infrastructure. Other potential barriers include the organisational level, the healthcare provider and patient factors. Some barriers are, however, potentially correctable, and the goal of the implementation of the recommendations for AS management is to translate evidence-based AS management recommendations into “real-life” practice which ultimately will lead to improved health status for patients with AS. It should be noted, however, that this study did not allow us to truly assess application of the ASAS-EULAR recommendations, but only the self-reported application. The possibility of a gap between self-declared application of recommendations, and their true application cannot be excluded.

The differences between agreement and application are of particular interest, since these differences reflect areas in which the doctors agreed with the recommendations, but reported they did not implement them. This section of the results also disclosed differences between countries, which is important since equality in access to healthcare should be a general goal in Europe The difference between agreement and application was high for recommendations 2, 4 and 9 and the overall country differences were highest in Italy and the UK. Recommendation 2 was deemed difficult to apply because of lack of time and this concern was especially reported by doctors in the UK. The country difference between agreement and application was pronounced for recommendation 9 (“anti-TNF”), and the largest discrepancy between agreement and the possibility of applying the recommendations was reported by doctors from the UK and the Czech Republic. The barriers cited by doctors refer to funding and administrative burden of anti-TNF. Thus, according to our results, there are inequalities in access to treatment and healthcare in Europe. The unfortunate situation for patients with AS in UK is not surprising since previous studies also have indicated that access to anti-TNF drugs for patients with rheumatoid arthritis is more limited in the UK than in, for example, the Netherlands and the Scandinavian countries.^13^

In conclusion, this project supported the dissemination of evidence-based recommendations for AS. However, the project also disclosed intercountry differences for conceptual agreement with the recommendations and implementation of the recommendations in clinical practice. The results show that inequalities exist in the provision of healthcare for patients with AS in Europe, even between countries who are members of the European Union.
